# Monitoring mRNA Half-Life in Arabidopsis Using Droplet Digital PCR

**DOI:** 10.3390/plants11192616

**Published:** 2022-10-05

**Authors:** Alexandre Boubegtitene, Rémy Merret

**Affiliations:** 1 CNRS-LGDP UMR 5096, 58 Avenue Paul Alduy, 66860 Perpignan, France; 2 Université de Perpignan Via Domitia, LGDP-UMR 5096, 58 Avenue Paul Alduy, 66860 Perpignan, France

**Keywords:** mRNA half-life, droplet digital PCR, Arabidopsis, ddPCR

## Abstract

mRNA decay is an important process in post-transcriptional regulation; in addition, it plays a crucial role in plant development and response to stress. The development of new tools to quantify mRNA decay intermediates is thus important to better characterize the dynamic of mRNA decay in various conditions. Here, we applied droplet digital PCR (ddPCR), a recent and precise PCR technology, to determine mRNA half-life in Arabidopsis seedlings. We demonstrated that ddPCR can correctly assess mRNA half-life from a wide variety of transcripts in a reproducible manner. We also demonstrated that thanks to multiplexing mRNA, the half-life of multiple transcripts can be followed in the same reaction. As ddPCR allows precise quantification, we proposed that this approach is highly suitable when a low amount of RNA is available; for the detection of many targets or for the analysis of lowly expressed transcripts.

## 1. Introduction

Gene expression is a key process that drives all metabolic and cellular processes required for organism development. In the cytoplasm, the regulation of mRNA levels controls the abundance of transcripts; and therefore, protein expression level. The abundance of an mRNA molecule is dictated by a fine balance between production and degradation processes.

mRNA decay is a highly conserved mechanism and was shown to be initiated by a deadenylation step [1]. After deadenylation, mRNA can enter in two distinct decay pathways; and being degraded from its 3’ extremity by the exosome complex or by its 5’ extremity by the exoribonuclease XRN1 (or XRN4 in plants) after a decapping step [1]. While these pathways are well characterized, their regulation and the respective contributions are not well understood. In addition to these canonical pathways, mRNAs can be also targeted to degradation while still associated with ribosomes. This pathway called co-translational mRNA decay was identified in many organisms such as yeast, humans or plants [2,3,4].

In a given cellular condition, each mRNA has an intrinsic lifetime at the end of which it is turned over. This half-life displays a wide range of stability varying from minutes to hours. For example, a recent non-invasive mRNA decay measurement approach reveals that in yeast, unstable transcripts have half-lives of less than 1 min; whereas stable transcripts have half-lives of more than 30 min [5]. In Arabidopsis, mRNA stability varies from a few minutes to several hours under normal conditions [6,7]. This intrinsic stability can also vary in response to a variety of stimuli, such as heat [8], cold [9] or osmotic stress [10]. Traditionally, mRNA half-lives are determined by following RNA degradation over time after blocking transcription using specific transcription inhibitors, such as cordycepin, α-amanitin or actinomycin D [11]; or by RNA labelling using a uridine analog [7]. RNA half-lives are next assessed by RNAseq and/or by qPCR. However, it could be difficult to validate several targets by qPCR on the same reaction mix as cDNA dilution is limited. Additionally, quantification of lowly expressed transcripts by qPCR requires a large quantity of material.

Droplet digital PCR (ddPCR) is a recent PCR method based on water–oil emulsion droplet technology that allows quantification of DNA molecules in an ultrasensitive manner. Prior to amplification, the template DNA (or cDNA) molecules are partitioned in individual droplets; allowing the measurement of thousands of independent amplification events within a single sample. Following PCR, each droplet is analyzed to determine the number of targets present in the original sample. This approach was already used to assess absolute quantification [12], copy number variation [13], rare mutation detection [14], gene rearrangement [15], pathogen detection [16] as well as DNA methylation [17]. Finally, multiplexing ddPCR assays using dsDNA-binding dye or labelled probes constitute an additional advantage permitting the detection of multiple targets in the same reaction [18].

As ddPCR allows precise and sensitive quantification, we developed and evaluated the efficiency of this technology to determine the transcripts’ mRNA half-life. We used *Arabidopsis thaliana* seedling as model and determined the mRNA stability of specific transcripts. We demonstrated that ddPCR assay allows a direct quantification of mRNA half-life for both stable and unstable transcripts in a reproducible manner. We also demonstrated that ddPCR allows a precise determination of the mRNA half-life of lowly expressed transcripts.

## 2. Results

### 2.1. Determination of Optimal Conditions for ddPCR Quantification

ddPCR slightly differs from conventional PCR or quantitative PCR. A reaction mix containing classical reagents for PCR amplification including a DNA dye is prepared. However, prior to amplification, a sample partitioning is performed using oil emulsion. Following PCR, each droplet is analyzed to determine the number of PCR-positive droplets (containing the target of interest) and PCR-negative droplets in the original sample. These data are then used to determine the concentration of the target in the original sample using Poisson statistics. This approach relies on a clear distinction between positive and negative droplets.

**Figure 1:**
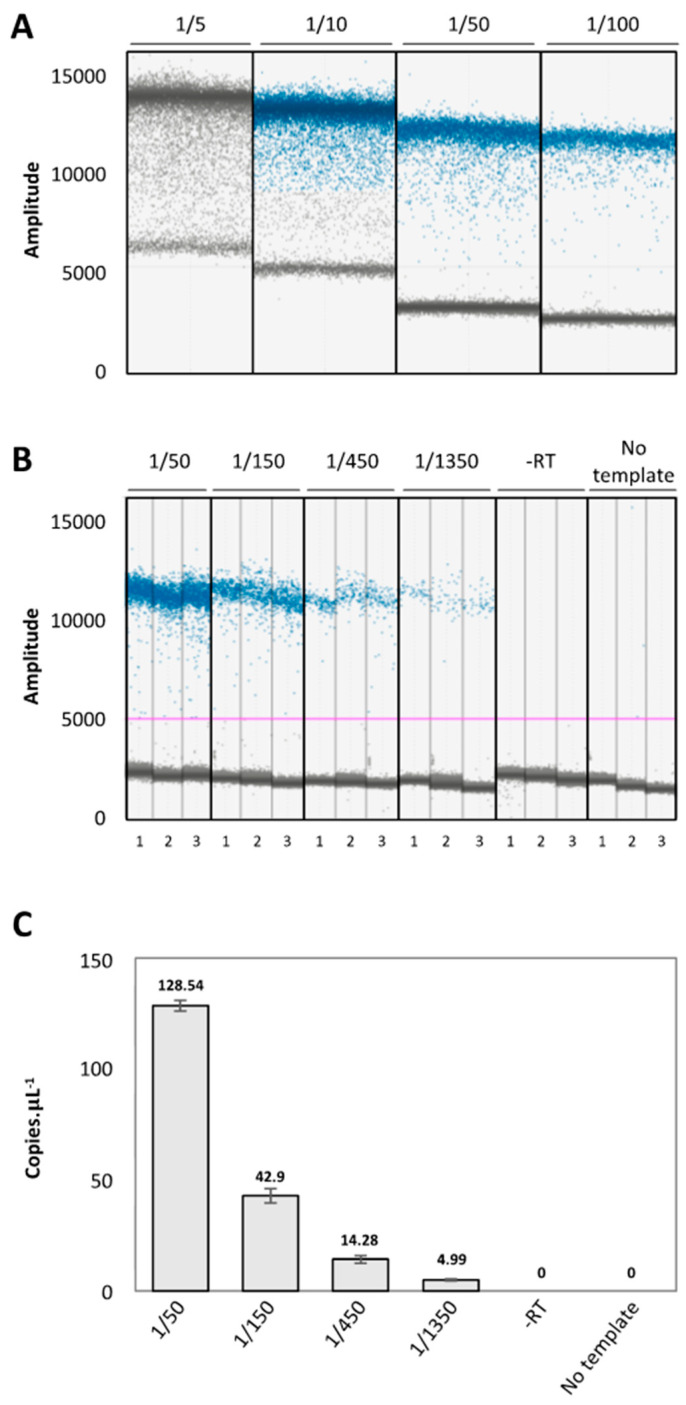
The importance of cDNA dilution for ddPCR quantification. (**A**). A ddPCR amplitude plot for At1G13245 according to the cDNA dilution factor. (**B**). A ddPCR amplitude plot for At1G13245 according to the cDNA dilution factor in each replicate. The blue and grey dots correspond, respectively, to the positive and negative droplets. The pink line corresponds to the threshold. (**C**). The number of copies per μL obtained in B. N = 3. Mean ± SD. The mean value is indicated on each bar.

Thus, prior to the determination of mRNA half-life by this approach, we determined the optimal conditions to clearly distinguish the positive and negative droplets. First, we designed primers that amplify short amplicons (between 50 and 250 pb) with a melting temperature (Tm) close to 60 °C. Primer specificity was first checked on gel by classical PCR amplification (Supplemental Figure S1). Next, we tested the impact of cDNA dilution on ddPCR quantification; and the discrimination between positive and negative droplets using primers against AT1G13245 (Figure 1A). Total RNA was extracted from 5-d-old seedlings and 500 ng was used for reverse transcription. cDNAs were then used for a serial dilution and analyzed by ddPCR. A five-fold dilution did not allow a discrimination between the positive and negative droplets. However, from ten-fold to hundred-fold dilution, positive and negative droplets can be generated; in addition, the interface between both populations increases with the dilution factor (Figure 1A). Since the goal of this study was to assess mRNA half-life with different ranges of stability, we decided to use fifty-dilution as a starting point for all downstream experiments. As mRNA stability can vary from minutes to hours, we tested if ddPCR can detect low concentration samples in a linear manner. To do so, we performed a serial dilution of cDNA from 50-fold to one 350-fold and used it as template for ddPCR (Figure 1B,C). We also included two negative controls: a sample without reverse transcriptase (-RT); and a sample without a PCR template (no template). The ddPCR data generated with AT1G13245 primers revealed good separation between the negative and positive droplets (Figure 1B). In both negative controls, no quantification was observed and the absolute concentration of the targets correlates perfectly with the applied 3-fold dilution factor (Figure 1C). Additionally, low variability was observed between the replicates; thus supporting the robustness of the approach. This approach allows us to determine the optimal conditions to correctly assess a wide range of mRNA half-life.

### 2.2. ddPCR Technology Allows an Accurate Determination of mRNA Half-Life

**Figure 2:**
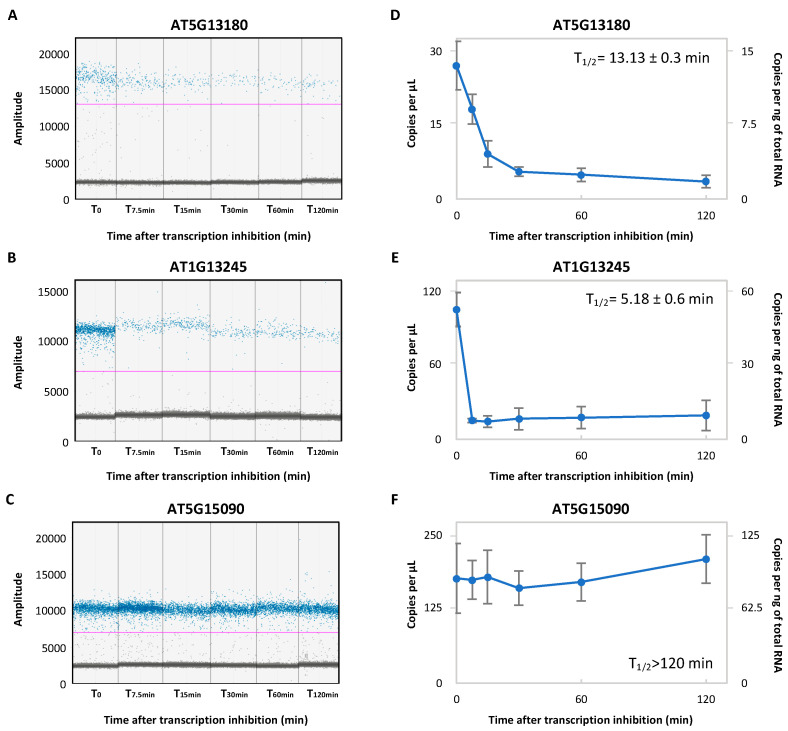
Monitoring mRNA half-life using ddPCR. A ddPCR amplitude plot for AT5G13180 (**A**), AT1G13245 (**B**) and AT5G15090 (**C**) during the transcription inhibition time course. The blue and grey dots correspond, respectively, to the positive and negative droplets. The pink lines correspond to the threshold; and were manually applied, respectively, at 13,000, 7000 and 7000 to AT5G13180, AT1G13245 and AT5G15090. (**D**–**F**). The number of copies per μL or per ng of the total RNA obtained in (**A**–**C**), respectively. N = 3 biological replicates. Mean ± SD.

In order to directly compare the mRNA half-life determined by ddPCR to the published mRNA half-life, we performed the same experimental design as in [6]. Arabidopsis 5-d-old seedlings were treated in vivo with a transcription inhibitor (cordycepin). As the median mRNA half-life in Arabidopsis is 107 min and because few transcripts present a half-life lower than 5 min [6], seedlings were collected 0, 7.5, 15, 30, 60 and 120 min after transcription inhibition in order to capture the unstable and stable transcripts. Total RNA was then extracted, and the same amount of material was used for reverse transcription. All the cDNA samples were then diluted 50-fold and subjected to ddPCR quantification. To demonstrate the validity of the approach, we decided to perform ddPCR quantification on three transcripts with different stabilities: AT5G13180 and AT1G13245 as unstable transcripts; and AT5G15090 as a stable transcript [6]. For each target, a clear distinction between the positive and negative droplets is observed for all conditions (Figure 2A–C).

We next repeated the analysis on three biological replicates and determined the number of targets along the time course (Figure 2D–F). For AT5G13180 and AT1G13245 described as unstable transcripts, a rapid decrease of copies number is observed; reaching a minimum at 30 min and 7.5 min, respectively, followed by a plateau up to 120 minutes (Figure 2D,E). Half-life determination gives reproducible results with low variability (AT5G13180, half-life: 13.13 ± 0.1 minutes; AT1G13245, half-life: 5.18 ± 0.5 min). For AT5G15090 described as a stable transcript, the number of copies remains relatively stable along the time course (Figure 2F). As no significant decrease is observed along the time course, half-life for this transcript was not determined and considered higher than 120 minutes. All together, these data support the prevalence of ddPCR for mRNA half-life determination.

### 2.3. Multiplexing Assay Allows Determination of mRNA Half-Life of Distinct Transcripts in a Single Reaction

**Figure 3:**
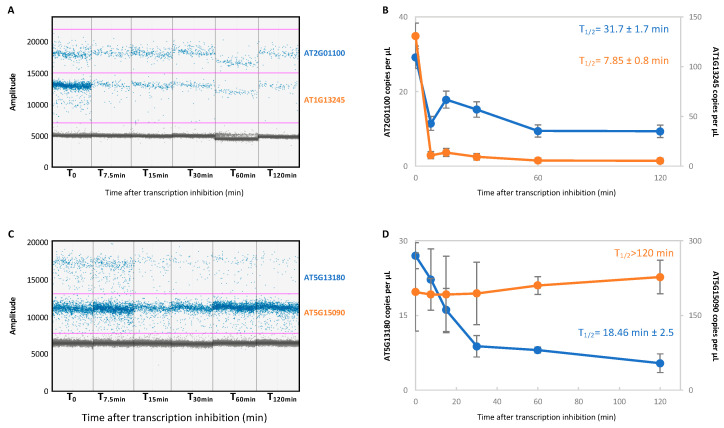
ddPCR allows monitoring of the mRNA half-life of 2 transcripts in the same reaction. (**A**). Multiplexed ddPCR target detection of AT1G13245 and AT2G01100 transcripts. Multiplexed detection (based on differences in amplicon lengths) was achieved by combining the AT1G13245 and AT2G01100 primers into a single reaction. The blue and grey dots correspond, respectively, to the positive and negative droplets. The pink lines correspond to the threshold and were manually applied at 7000 and 15,000 to AT1G13245 and AT2G01100, respectively. (**B**). The number of copies per μL obtained in (**A**). (**C**). Multiplexed ddPCR target detection of AT5G15090 and AT5G13180 transcripts. The pink lines correspond to the threshold and were manually applied at 7000 and 13,000 to AT5G15090 and AT5G13180, respectively. (**D**). The number of copies per μL obtained in (**A**). N = 3 biological replicates. Mean ± SD.

In addition to precise and sensitive quantification of targets, ddPCR can be used to quantify different targets in a single reaction thanks to a multiplexing assay. Multiplexing can be achieved by TaqMan probes with distinct fluorophores or by single fluorophore using EvaGreen dye. In fact, since the level of EvaGreen fluorescence is dependent on the amplicon length, two targets with distinct amplicon lengths can be quantified in a single reaction. As TaqMan probes are quite expensive, we decided to test if an EvaGreen multiplexing assay could allow the determination of the mRNA half-life of two transcripts in a single reaction. We designed a pair of primers targeting two transcripts with distinct amplicon length (113 nt for AT2G01100 and 61 nt for AT1G13245, Supplemental Figure S1). Each pair of primers was added in the reaction mix and droplets were analyzed along the time course experiment (Figure 3A,B). Three distinct populations of droplets are observed: negative droplets (grey dots with an amplitude lower than 5000); AT1G13245 positive droplets (blue dots with an amplitude between 5000 and 15,000); and AT2G01100 positive droplets (blue dots with an amplitude higher than 15,000).

Droplets quantification revealed distinct and reproducible mRNA half-lives for both transcripts (Figure 3B). AT1G13245 presents a half-life slightly different than that observed in the simplex assay, but in the same range (13.13 min in the simplex assay compared to 18.46 min in the multiplex assay). We also noticed that the standard deviation slightly increases in the multiplex assay. We repeated a multiplexed assay with AT5G13180 and AT5G15090 primers (Figure 3C,D). Here again, three distinct populations are observed and half-lives similar to the simplex assay were obtained. These data suggest that multiplexing does not greatly affect mRNA half-life determination. 

### 2.4. Characterization of mRNA Half-Life of Lowly Expressed Transcripts

**Figure 4:**
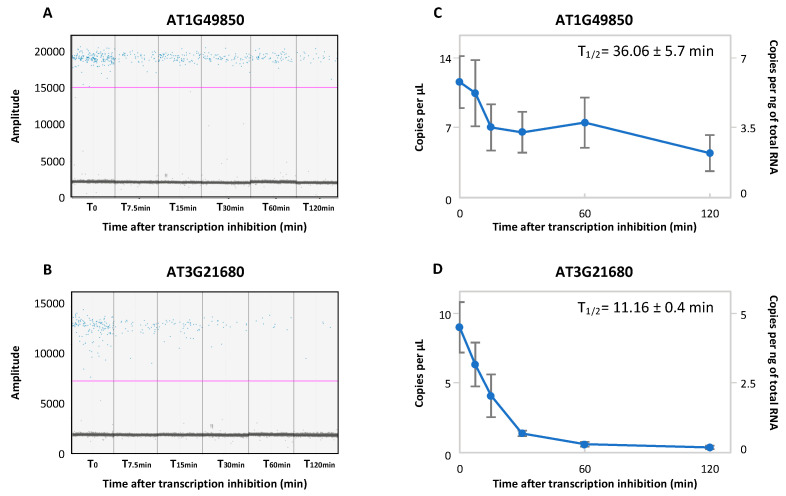
ddPCR allows monitoring of the mRNA half-life of lowly expressed transcripts. A ddPCR amplitude plot for AT1G49850 (**A**) and AT3G21680 (**B**) during the transcription inhibition time course. The blue and grey dots correspond, respectively, to the positive and negative droplets. The pink lines correspond to the threshold and were manually applied, respectively, at 15,000 and 10,000 to AT1G49850 and ATG321680. (**C**,**D**). The number of copies per μL or per ng of the total RNA obtained in (**A**) and (**B**), respectively. N = 3 biological replicates. Mean ± SD.

The main advantage of ddPCR is its ability to allow low-copy target detection compared to qPCR. We thus tested if ddPCR allows mRNA half-life determination of lowly expressed transcripts. We chose two transcripts (AT1G49850 and AT3G21680) identified as lowly expressed at the 5-d-old seedling stage [6] and quantified their level across the time course experiment (Figure 4). Both targets present a clear discrimination between the positive and negative droplets (Figure 4A,B). As expected, both transcripts were identified with less than 15 copies.μL^−1^ at T_0_ (Figure 4C,D). Although both transcripts are reported as unstable, ddPCR allows a precise quantification across the time course experiment. Both transcripts can be correctly quantified with, respectively, a half-life of 36.06 ± 5.6 and 11.16 ± 0.35 min.

## 3. Discussion

For many years, mRNA decay appears to be an important process for plant development and response to stress [3,8,19,20,21,22]. The development of accurate methods to quantify decay intermediates is crucial to understand the importance of mRNA turnover in gene expression regulation. In this study, we tested if ddPCR can be used as an additional tool to determine mRNA half-life in Arabidopsis. 

To do so, we performed a transcription inhibition time course experiment using an Arabidopsis seedling as model, followed by target quantification using ddPCR technology. We first demonstrated that the optimization of cDNA dilution is crucial prior to analysis to clearly discriminate positive and negative droplets and to allow a precise targets quantification. This kind of optimization was already proposed in many studies and was important to prevent droplets saturation; reverse transcription inhibitor effects; or low discrimination between positive and negative droplets [23,24,25,26]. 

After optimization, we demonstrated that ddPCR allows a precise quantification of unstable and stable transcripts independently of the target expression level. The half-lives reported here are similar to those already published. In fact, a genome-wide mRNA decay analysis on Arabidopsis seedlings was recently published [6]. For example, AT5G13180 and AT1G13245 were reported as unstable transcripts with a respective half-life of 11.83 and 29.6 min. For these transcripts, the ddPCR approach reveals similar half-lives (Figure 2). For AT1G13245, ddPCR reveals that this transcript is unstable as published; but with a lower half-life. Precise determination of the mRNA half-life on lowly expressed transcripts was also observed by ddPCR and similar to the published data (AT1G49850 half-life: 36.06 min by ddPCR, 27.07 min in [6]; AT3G21680 half-life: 11.16 min by ddPCR, 11.23 min in [6]). All together, these comparisons demonstrate that the ddPCR approach reveals reproducible and relevant mRNA half-lives. This approach is thus suitable for mRNA decay analysis and can be easily adapted to other model organisms. To our knowledge, this approach was never used in any model organisms and provides a new tool for the RNA biology field. 

The determination of mRNA half-life by ddPCR presents several advantages compared to other techniques, such as RT-qPCR or Northern blot. First, contrary to qPCR, ddPCR allows an absolute quantification; and is not subjected to primer efficiency, which highly affects qPCR quantification [27]. A standard curve essential in qPCR is not necessary for ddPCR; this significantly reduces the number of samples to analyze. Secondly, more targets can be analyzed by ddPCR as template dilution is more important than qPCR. This aspect could be particularly interesting when a limited amount of RNA is available or to limit the subsampling effect that can occur when multiple reverse transcriptions are performed on the same RNA sample [28]. Thirdly, we showed that ddPCR multiplexing allows the determination of the mRNA half-life of multiple targets in the same reaction; making the approach suitable for direct comparison of multiple mRNA half-lives in the same PCR reaction. Finally, as ddPCR is highly sensitive, half-lives of lowly expressed transcripts can be also determined in a significant manner on a small amount of material.

## 4. Materials and Methods

### 4.1. Growth Condition and Transcription Inhibition

Analyses were carried out with Columbia-0 line as wild type. Plantlets were grown during 5 days on a synthetic Murashige and Skoog medium (Duchefa) containing 1% Sucrose and 0.8% plant agar at 22 °C under a 16-h-light/8-h-dark regime. Transcription inhibition was performed as previously described in [6]. Seedlings were collected at 0, 7.5, 15, 30, 60 and 120 min in three biological replicates. 

### 4.2. RNA Extraction and Reverse-Transcription

Total extraction was performed using a Monarch total RNA miniprep kit (New England Biolabs). DNase treatment was performed during RNA extraction using the manufacturer’s instructions. Reverse transcription was conducted on 500 ng of RNA in a final volume of 20 μL using the Superscript IV RT-system and a random primer (Fisher Scientific). cDNAs were then diluted 50-fold and stored at −20 °C.

### 4.3. Primer Design

Primers used for ddPCR were designed using Primer3 software with the following features: Tm between 59 and 61 °C; amplicon size between 50 and 250 bp; and primer length between 19 and 21 nt. Primers are listed in Table S1. 

### 4.4. ddPCR Reaction

ddPCR was performed using QX200 ddPCR Evagreen Supermix (Biorad), following the manufacturer’s instructions. A reaction mix was prepared containing 1X QX200 ddPCR EvaGreen Supermix, 100 nM of forward primer, 100 nM of reverse primer and 4.4 μL of cDNA in a final volume of 22 μL. Then, 20 μL of this mix was used for droplets generation using 70 μL of QX200 droplet generation oil (Biorad). Subsequently, 40 μL of the mixture was transferred in a thermocycler for amplification using the following program: 5 min at 95 °C, followed by 40 cycles of 30 s at 95 °C; and 1 minute at 60 °C (ramp rate 2 °C/second), followed by 5 min at 4 °C and 5 min at 90 °C. The mixture was then kept at 4 °C for l hour to overnight to stabilize the droplets. Data acquisition and analysis were performed using a QX200 droplet reader and QuantaSoft software, respectively. A threshold was manually applied based on the interface between positive and negative droplets. For each target, the same threshold was applied for all the tested conditions. The concentration (copies.μL^−1^) in each condition was determined using the merged analysis setting. The standard deviation (SD) was calculated using the following equation:

SD = (CI_max_ − CI_min_)/(2 × 1.96) (1)

where CI_max_ and CI_min_ indicate the upper and lower limits of the 95% confidence interval (called “TotalConfMax” and “TotalConfMin” in QuantaSoft Software). For the duplex assay, the design was similar to the simplex assay except with each pair of primers used at 100 nM. As reverse transcription was conducted on standardized 500 ng of total RNA in 20 μL (25 ng μL^−1^) and as ddPCR quantification was performed using 4 μL of 50-fold diluted cDNA (=2 ng of total RNA), the concentration can be expressed in copies per ng of the total RNA.

### 4.5. mRNA Half-Life Determination

To determine the mRNA half-life, the constant of mRNA decay (K_decay_) was first determined using the following equation:

K_decay__=_= −ln(C_t_/C_0_)/t (2)

where C_t_ and C_0_ represent, respectively, mRNA quantity at time t and time 0. Next, the following equation was used to determine mRNA half-life (t_1/2_):

t_1/2_ = ln2/K_decay_ (3)

## Data Availability

Not applicable.

## Supplementary Materials

The following supporting information can be downloaded at: https://www.mdpi.com/article/10.3390/plants11192616/s1, Figure S1: Validation of primers specificity; Table S1: Primers used in this study.
