# Supplementary material for: Monitoring mRNA Half-Life in Arabidopsis Using Droplet Digital PCR
Source: Plants (Basel). 2022 Oct 5;11(19):2616. doi: 10.3390/plants11192616 (PMC9571659; doi:10.3390/plants11192616)
Supplement: Supplementary file 1 [file plants-11-02616-s001.zip › FigureS1.pdf]

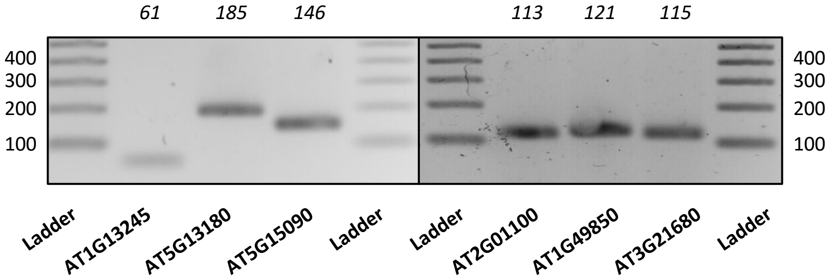

Supplemental Figure S1 : Validation of primers specificity. Primers specificity was checked by PCR amplification followed by gel migration. Predicted size is indicated in bp.
