# Supplementary material for: Monitoring mRNA Half-Life in Arabidopsis Using Droplet Digital PCR
Source: Plants (Basel). 2022 Oct 5;11(19):2616. doi: 10.3390/plants11192616 (PMC9571659; doi:10.3390/plants11192616)
Supplement: Supplementary file 1 [file plants-11-02616-s001.zip › TableS1.pdf]

Table S1 : Primers used in this study.

Gene_ID	Amplicon size (nt)	Forward primer 5'-3'	Reverse primer 5'-3'
AT5G13180	185	AGAGCCGGTAACAAGAACGA	GAGCTCGGCAAAAGGTTCAA
AT1G13245	61	TGGAGGGAGAAGAGTCGTGA	AGAATCAAACCCGATCCGCC
AT5G15090	146	TACCTTTGATGAGCCTGCCC	TGACAATCGGGGTAGCTGTG
AT1G49850	121	CGACACCACTCACACCATCA	GGTGAAAGAAGCGTCGGAGA
AT3G21680	115	GCCTAAGACACGACAATGGC	TCACTTTCTTCTTCCCCACGG
AT2G01100	113	GCGAGGCAAATATCACAGGC	ACTCAGCCAAACGATGCCTT
